# Assessing rare diseases prevalence using literature quantification

**DOI:** 10.1186/s13023-020-01639-7

**Published:** 2021-03-20

**Authors:** Shourick Jason, Wack Maxime, Jannot Anne-Sophie

**Affiliations:** 1 Department of Medical Informatics, Hôpital Européen Georges Pompidou, AP-HP, 20 Rue Leblanc, 75015 Paris, France; 2 INSERM, Centre de Recherche des Cordeliers, UMRS 1138, Université de Paris, Université Sorbonne Paris Cité, Paris, France

**Keywords:** Rare diseases, Bibliometrics, Prevalence

## Abstract

**Introduction:**

Estimating the prevalence of diseases is crucial for the organization of healthcare. The amount of literature on a rare pathology could help differentiate between rare and very rare diseases. The objective of this work was to evaluate to what extent the number of publications can be used to predict the prevalence of a given pathology.

**Methods:**

We queried Orphanet for the global prevalence class for all conditions for which it was available. For these pathologies, we cross-referenced the Orphanet, MeSH, and OMIM vocabularies to assess the number of publication available on Pubmed using three different query strategies (one proposed in the literature, and two built specifically for this study). We first studied the association of the number of publications obtained by each of these query strategies with the prevalence class, then their predictive ability.

**Results:**

Class prevalence was available for 3128 conditions, 2970 had a prevalence class < 1/1,000,000, 41 of 1–9/1,000,000, 84 of 1–9/100,000, and 33 of 1–9/10,000. We show a significant association and excellent predictive performance of the number of publication, with an AUC over 94% for the best query strategy.

**Conclusion:**

Our study highlights the link and the excellent predictive performance of the number of publications on the prevalence of rare diseases provided by Orphanet.

## Introduction

The European Union Regulation on Orphan Medicinal Products defines a disease as rare if it affects no more than 1 person in 2000 in the European population [1], making epidemiologic data and more specifically prevalence data a key point to develop health care policies for patients with rare diseases. Prevalence data are needed to support the “orphan” designation for a drug, either investigational or already approved, prevalence and burden factors often being considered by policy-makers in taking decisions about the allocation of resources for biomedical research.

Furthermore, even though each disease is rare in and of itself, patients with rare diseases are common, accounting for 3.5–5.9% of the Worldwide population [2] which equates to 263–446 million persons. This public health issue is even aggravated by the high heterogeneity of rare diseases, in aetiology (genetic, immunologic, and cancerous) but also in prevalence. Indeed rare diseases prevalence can range from locally rare diseases but frequent worldwide, or even epidemic in some locations (such has Dengue, Zika virus or Ebola in Europe), to diseases with only one case reported.

Due to these large discrepancies in prevalence, the question about a rare disease might often not be “Is it rare?” but “How rare is it?”.

Despite its importance, the absence of prevalence data can be a common situation in rare diseases. In these situations, the number of cases or families documented in the medical literature is the only epidemiologic information available and is therefore used as an indication of rarity.

Orphanet carries out a systematic review of the literature in order to estimate the prevalence and incidence of rare diseases. Among the 9408 clinical entities (groups of diseases, disorders, and sub-types) contained in the Orphanet database, epidemiological data is available for 5949 (63%) of them. In the June 2018 report on bibliographic data published by Orphanet, data was available for 4336 diseases including 860 diseases with precise prevalence information, 351 with precise incidences information, 2754 diseases with number of published cases information, and 376 cases with number of published families information [3].

Therefore, there is no estimation of disease prevalence for more than a third of rare disease while this information is crucial. The objective of this study is to provide a method to estimate rare disease prevalence without epidemiological study when it is lacking in public database. To this end, we hypothesise that prevalence could be inferred using the amount of literature on a given disease.

Bibliometric data on rare diseases are scarce but some publications showed that the majority of publications for rare diseases are case reports, rarely reporting more than one case at once, and spread on numerous years and languages [4–7].

Furthermore, literature searches for rare diseases are rendered more difficult by the lack, or only partial mapping, of terminology concepts for some of these diseases. For example, MeSH terms were scarce for rare diseases before 2010 [8]. Among the 9408 clinical entities in Orphanet, only 4833 are mapped within the UMLS, 1753 within Mesh, and 4491 within OMIM [9].

The aim of this study was first to validate this hypothesis of a strong association between the amount of literature for a given disease and its prevalence, assessed manually by Orphanet. As there is no gold standard for the amount of literature for a given disease, we compared several automatized, terminology-based search query strategies on a large set of rare diseases. We then explored the discriminating performance of each query strategy to predict the disease’s class or prevalence with the purpose of predicting prevalence with automatic query strategy searches.

## Methods

### Pubmed overview

Pubmed is the most commonly used database for bibliographic research in medicine. It comprises more than 30 million citations for biomedical literature, more than 25 million from MEDLINE. Each MEDLINE entry is manually annotated with a list of MeSH terms. MeSH terms are organised in two ways: on one hand in Concept and Supplementary concepts, which refers to a group of exact terms and all exact synonyms, containing one preferred term; and on the other hand in Descriptor in which the preferred term subsumes the other. PubMed can be queried using multiple terms connected by Boolean operators, and search terms can be specified with Pubmed operators in brackets allowing the user to specify the field in which the term is to be queried [10].

In this article we used 4 Pubmed operators:

- [tiab] where the term is a free text keyword, searched for only in the abstract and title fields of PubMed citations,
- [tw] where the term is a free text keyword, searched for in multiple fields of PubMed citations (title, abstract, MeSH heading, other keywords etc.),
- [mh] where the term is a MeSH descriptor all the terms it subsumes are searched for in MeSH headings,
- [nm] where the term is a MeSH supplementary concept with all the terms it subsumes, searched for in MeSH headings.

Furthermore we used the complement operator “:noexp”, placed in the bracket after the Pubmed operator. It allows for the search of only the preferred MeSH term but ignores the terms it subsumes therefore allowing for a choice in the desired granularity.

All combinations of Pubmed operator are explained in Table 1.

**Table 1:** Pubmed operators used in the study

Operator	Meaning
[tiab]	The term is considered as a free text keyword and searched for in abstract and title fields of PubMed citation
[tw]	The term is considered as a free text keyword and searched for in multiple fields of PubMed citation (title, abstract, MeSH indexing, other keywords etc.)
[mh]	The term, a MeSH descriptor, and all the terms it subsumes, are searched for in MeSH indexing
[mh:noexp]	The term, a MeSH descriptor is searched for in MeSH indexing,, but the terms it subsumes are ignored
[nm]	The term, a MeSH supplementary concept and all the terms it subsumes, are searched for in MeSH indexing
[nm:noexp]	The term, a MeSH supplementary concept is searched for in MeSH indexing,, but the terms it subsumes are ignored

### Orphanet epidemiological data extraction

We used the data on rare diseases epidemiology published by Orphanet in January 2019 [9]. This bibliographic research has been conducted over different sources: Registries; National and International health institutes; Medline using the following search query strategy: “Disease name” AND Epidemiology[MeSH:NoExp] OR Incidence[Title/abstract] OR Prevalence[Title/abstract] OR Epidemiology[Title/abstract]; Medical texts; grey literature and reports; and Orphanet collaborating experts.

Orphanet provides class prevalence with the location where the prevalence was evaluated. We used all diseases for which the class of prevalence was available and to avoid local discrepancies in the rarity of diseases we used diseases where prevalence was assessed worldwide.

As the database contains diseases with prevalence higher than 1 person per 2000 (European definition of rare disease), we censored diseases for which prevalence class was more than 1 person per 1000 to comply at best with the European definition of rare diseases. The remaining prevalence classes were: < 1/1,000,000, 1–9/1,000,000, 1–9/100,000 and 1–9/10,000.

### Orphanet cross-classification and proposed automated mapping for missing Orphanet cross-classification

Orphanet provides its own controlled vocabulary organised by name of the disease (referred as Orpha term in the rest of the article) and exact synonyms (referred as Orpha Synonyms).

Orphanet provides a cross referencing of Orpha, MeSH, and OMIM terms, which we used when available. When no cross referencing was available between Orpha terms and MeSH terms, but available between Orpha and OMIM, and between OMIM and MeSH, we applied the transitive relation to map Orpha and MeSH terms through OMIM. The mapping between MeSH and OMIM terms was created through the extraction of all exact synonyms between the OMIM and MeSH terms in the UMLS metathesaurus.

### Publication numbers extraction

We used three different Pubmed query strategies quantifying the amount of literature on a given disease.

For the two query strategies we created, we excluded all acronyms from Orpha and Mesh terms, as those might refer to multiple diseases (such as “FACE” for Fanconi anemia). The same pre-processing step could not be applied to OMIM terms as all terms are provided in uppercase.

The last query strategy we used is the one described by Griffon et al*.*, from which we excluded the Human Phenotype Ontology (HPO) terms, as HPO contains phenotypes and not diseases [11].

The query strategies are described in Table 2. Briefly, the first query strategy contains Orpha and Mesh terms while the second only contains Orpha terms. Orpha terms and synonyms are queried as free text in titles and summary with the [tiab] operator whereas MeSH terms are queried as free text with the [tiab] operator and as MeSH indexing using the [mh]:noexp (for concept) or [nm]:noexp (for supplementary concept] operators.

**Table 2:** Description of query strategies

	Query Strategy (QS) 1	Query Strategy (QS) 2	Query Strategy (QS) 3 (Griffon et al.)
Orpha	[tiab]	[tiab]	[tw]
Orpha Synonyms	[tiab]	[tiab]	[tw]
If the disease has a Mesh Descriptor (Mesh prefered term)	[mh:noexp]	-	[mh]
If the disease has a Mesh Supplementary Concept (Mesh prefered term)	[nm:noexp]	-	[nm]
If the disease has a Mesh Concept	[tiab|	-	[tw]
If the disease has Mesh Synonyms (Mesh non prefered term)	[tiab]	-	[tw]
If the disease has an OMIM term	-	-	[tw]
If the disease has an OMIM Synonyms (OMIM non prefered term)	-	-	[tw]

### Statistical analysis

A non-parametric Kruskal–Wallis test was performed to compare publication number distributions of each prevalence class.

The performance of the 3 query strategies to discriminate between classes was then assessed as follows.

Prevalence was used as the dependant variable and publication number as the independent variable to produce a ROC curve. The best publication number threshold, defined as the threshold maximizing Youden’s index (sum of sensitivity and specificity minus one) was then selected. Confidence intervals for sensitivity, specificity, and AUC were estimated using bootstrapping. Because prevalence is discretized in ordinal classes, for each query strategy one ROC curve was used to predict: < 1/1,000,000 versus others, < 100,000 versus others, < 10,000 versus others.

All data extraction, number of publications and statistical analyses were conducted using R version 3.5.1. ROC curves and related analyses used the pROC package [12].

## Results

Among all 3128 entities, 2970 had a prevalence class of < 1/1,000,000, 41 had a prevalence of 1–9/1,000,000, 84 had a prevalence of 1–9/100,000, and 33 had a prevalence of 1–9/10,000.

### Query strategy 1

For the first query strategy, the number of retrieved publications ranged from 0 to 100,000 (maximum retrievable using the pubmed E-utilities API), with 1423 entities with no publication retrieved, and one entity presenting a number of publication over 100,000 (Obesity due to pro-opiomelanocortin deficiency). We believe that this error is due to the mapping between Orpha and OMIM terms, the linked OMIM term being “Obesity” which then links to the broad MeSH descriptor “Obesity”. When OMIM and MeSH term were excluded from this query 30 publication were retrieved.

### Query strategy 2

For the second query strategy, the number of retrieved publications ranged from 0 to 30,507, with 1761 entities with no publication retrieved, and no entity with more than 100,000 publications retrieved. We hypothesised that this higher number of diseases without any publication retrieved with this query strategy is due to the more limited number of keywords used. To test this hypothesis we calculated the correlation (Spearman’s test) between the difference in number of publication between query strategy one and two, and the difference in number of key words between query strategy one and two. We obtained a high correlation of 0.61 [0.587, 0.631], *p* value << 0.001 confirming our hypothesis.

### Query strategy 3

Finally, for the third query strategy, the number of retrieved publications ranged from 0 to 100,000, with 1063 entities with no publication retrieved, and 9 with more than 100,000 publication retrieved. We believe that the higher number of diseases with over 100,000 publication might be due to the presence of unspecific acronyms in OMIM terms. Indeed eight of the nine diseases of interest were represented with OMIM terms such as “FACE” for Fanconi anemia, or “SD” for Free sialic acid storage disease; the ninth disease being Obesity due to pro-opiomelanocortin deficiency, as previously explained.

### Association between the number of publications and the prevalence rate

For each query strategy, the Kruskal–Wallis test showed a very significant difference of number of publications in each prevalence class (Table 3), with p value for all query strategies under < 0.001.

**Table 3:** Number of publication in each prevalence class

	Prevalence: < 1/ 1,000,000		Prevalence: 1–9/1,000,000		Prevalence: 1–9/100,000		Prevalence: 1–9/10,000		
	Median	IQR	Median	IQR	Median	IQR	Median	IQR	*p*
Query strategy 1	1	18	635	1486	971	3151	3085	5349	< 0.001
Query strategy 2	0	10	351	684	511.5	1567	1285	4411	< 0.001
Query strategy 3	4	40.25	1291	5183	1662.5	6560	3271	15,283	< 0.001

**Fig. 1:**
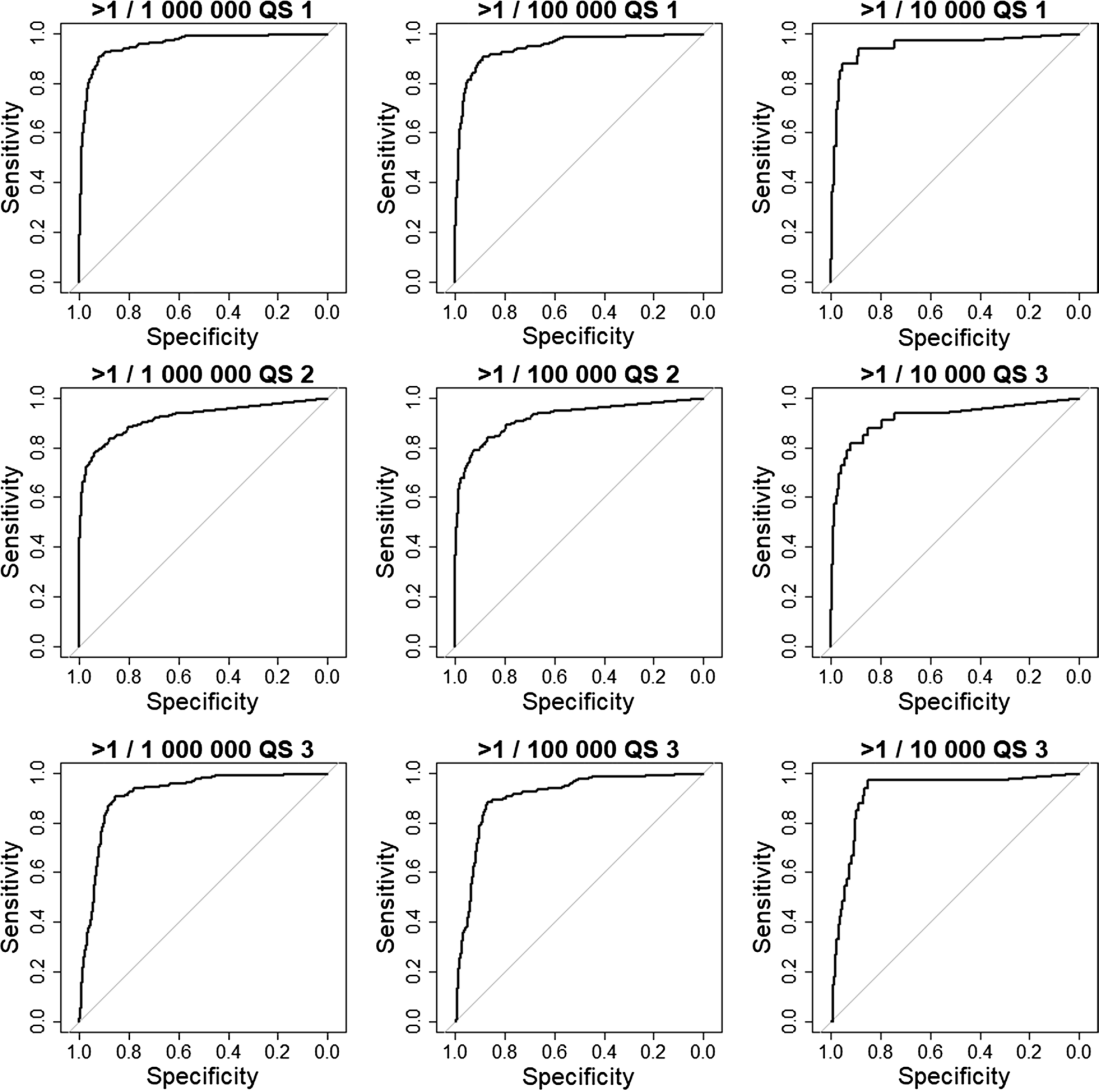
ROC curves for each query strategy and each prevalence category. Footnote: QS Query strategy

All ROC curves are presented in Fig. 1. AUC sensitivity and threshold using Youden’s index are presented in Table 4.

**Table 4:** Discriminating capacities of each query strategy

Prevalence	Number of diseases	Treshold	Sensibility % [IC 95%]	Specificity % [IC 95%]	AUC% [IC 95%]
Query strategy 1					
< 1/1,000,000	2970				
1–9/1,000,000	41	104.5	90.5 [85.4, 94.9]	91.9 [90.9, 92.8]	95.6 [93.9, 97.3]
1–9/100,000	84	74.5	90.6 [84.6, 95.7]	88.7 [87.5, 89.8]	94.5 [92.2, 96.8]
1–9/10,000	33	487	87.9 [75.8, 97]	95.2 [94.4, 95.9]	94.9 [90.2, 99.6]
Query strategy 2					
< 1/1,000,000	2970				
1–9/1,000,000	41	74.5	78.5 [72.2; 84.8]	93.7 [92.8, 94.5]	91.9 [89.1, 94.8]
1–9/100,000	84	74.5	78.6 [70.9; 85.5]	92.7 [91.7, 93.6]	92.2 [89.1, 95.2]
1–9/10,000	33	93.5	81.8 [69.7; 93.9]	92.1 [91.1, 93]	92 [86.1, 98]
Query strategy 3					
< 1/1,000,000	2970				
1–9/1,000,000	41	147.5	90.5 [86.1, 94.9]	85.4 [84.1, 86.7]	91.4 [89.4, 93.5]
1–9/100,000	84	229	88 [82.1, 94]	86.8 [85.6, 88.1]	90.3 [87.7, 92.9]
1–9/10,000	33	229	97 [90.9, 100]	84.9 [83.7, 86.1]	91.8 [87, 96.6]

The first query strategy presented the best discriminating abilities (Sensitivity and specificity are given for best Youden’s index).

The AUC to distinguish between < 1/1,000,000 and 1/1,000,000 to 9/10,000 was 95.6 [93.9, 97.3], sensitivity was 90.5 [86.1, 94.9], and specificity 91.9 [90.9, 92.9].

The AUC to distinguish between 1 to 9/1,000,000 and 1/100,000 to 9/10,000 was 94.5 [92.2, 96.8], a sensitivity of 90.6 [85.5, 95.7] and a specificity of 88.7 [87.5, 89.8].

Finally the AUC to distinguish between 1–9/10,000 and < 1/10,000 was 94.9 [90.5, 99.6] with a sensitivity of 87.9 [75.8, 96.9] and a specificity of 95.1 [94.4, 95.9].

Overall, the second query strategy, while having specificity close to the first query strategy, had worse sensitivity. On the contrary, the third query strategy while it had a close to or even better sensitivity than the first query strategy, had a lower specificity.

## Discussion

We demonstrated the strong predictive value of literature volume on the estimation of prevalence in rare diseases. As there is no gold standard to assess the volume of literature for a given disease, we tested 3 different query strategies sourcing from different ontologies and indexes, using both controlled vocabularies and free search terms, the third query strategy was published by Griffon et al*.* to perform exhaustive literature searches on any given rare diseases, based on four rare diseases terminologies: MeSH, OMIM, HPO, and Orphanet. They showed no significant differences in precision for query strategies based on literature queries compared to manual queries created by Orphanet experts. However this query strategy was only tested on 30 diseases [11].

Overall all the query strategies performed well, especially to differentiate rare diseases form very rare diseases (< 1/1,000,000 vs other). However, one of our query strategies was consistently better, for two reasons. First, it ignored acronyms that can lead to non-accurate free research terms, such as the Facial dysmorphism-anorexia-cachexia-eye and skin anomalies syndrome that is referred to as “FACES” or “FACES syndrome”. Second, it made use of the maximum available search terms without relying on acronyms, and emphasised the use of the MeSH controlled vocabulary, allowing for more articles to be identified through Pubmed keyword indexing.

At face value, this type of result can seem surprising because the amount of literature is influenced by many other factors. The link between prevalence of a diseases and amount of medical literature has not been widely studied. However, a previous bibliometric study of neurological diseases had shown that more articles were devoted to common diseases than to rare diseases, but in this study the link with the prevalence of rare diseases was not further explored [13]. Different other factors might influence the amount of literature. One of them is the effort done by some country, and some patients’ association, to push forward research on some specific kind of disease. In the European Union, the funding of research has been shown as an important factor influencing the amount and the impact factor of publications on rare diseases produced by a country [14], whereas another study showed that gross domestic product was only a modest predictor of publication numbers in public health [15]. We can imagine that diseases with high prevalence in rich countries receive more attention. Indeed neglected tropical diseases have a high prevalence worldwide but remained underfunded [16]. This correlation between the place were a disease is more prevalent and its importance in research programs loosens the link between worldwide disease prevalence and amount of literature. Publications amount also depends on the disease distribution: it has also been shown that for diseases with a high concentration in prevalence, such as Behçet’s disease, the countries where it is the most prevalent might not be the biggest publication sources [5]. Another institutional factor of importance is the development of referring centres, structures of high expertise on rare diseases both clinically and scientifically, and their ability to form collaboration on a national level [7, 17, 18]. These efforts can be loosely correlated with actual disease prevalence.

Some characteristic of the diseases themselves can also influence the amount of publications. The severity of the disease, often high in rare diseases, can mitigate the prevalence effect [19]. The presence of industrial interests such as in anaplastic large cell lymphoma of the breast also lead to more publication [20]. Some rare diseases such as monogenic obesity due to leptin-melanocortin pathway anomaly have also been of special interest as understanding this specific pathway might lead to therapeutic innovations or etiologic insights in other, more common, forms of obesity [21]. Furthermore, previous bibliographic articles on rare diseases have reported that rare diseases publications stems rarely from international collaborations and are likely to be published in low impact journals [4]. Therefore, the amount of literature on a rare disease also depends on peer review process and editorial policies.

The publication bias that favours studies presenting positive results might also have a role in the correlation between the rarity of a disease and the amount of publication [22]. However, this correlation might decrease the number of publication for the rarest diseases. Indeed the rarest a disease is, the less amount of patients a study is susceptible to include, therefore raising the chance of negative studies. Furthermore most rare diseases medication are repurposed one’s which might have a lower rate of success than specifically developed ones [23].

### Our methodology

The link between disease prevalence and amount of literature appears as natural because the amount of literature depends on the number of cases available for research. But it is the first time that the size effect of such a link is deciphered and that not only an association but a high predictive value is demonstrated. One consequence of this link is the lack of knowledge on very rare diseases despite international efforts. Our work has a high interest for public heath purposes. The proposed query strategy allows the classification of rare diseases prevalence based on publically available information, therefore at no cost. This can also be useful to help forecast health care needs for rare diseases or groups of rare diseases for which no other prevalence information is available. It can also be used as a worldwide reference tool when analysing empirical point prevalence in a given region or country.

The main limitation of this work is due to the repartition of diseases among prevalence classes. The vast majority of diseases belong to the group of very rare diseases. Therefore, the three query strategies presented thresholds that did not increase with prevalence class (Table 4).

However, the identification of very rare diseases versus other rare diseases is what is important for public health purpose and this is where our method performs best.

The second limit is the presence of cross-classification leading to a very high amount of publication on some diseases, these errors can however easily be spotted and recognised and present no threat if the results are interpreted disease by disease. Another source of prevalence class error is the lack of ontologies mapping, but this can also be partially overcome through checking.

## Conclusion

This study provides, for the first time to our knowledge, a way to assess worldwide prevalence of rare diseases at no cost using bibliometric indicators, offering valuable perspectives for public health applications.

## Data Availability

The datasets generated and analysed during the current study are available from the corresponding author on request.
