# Correction to: Assessing rare diseases prevalence using literature quantification

**DOI:** 10.1186/s13023-021-01854-w

**Published:** 2021-05-10

**Authors:** Jason Shourick, Maxime Wack, Anne-Sophie Jannot

**Affiliations:** 1 Department of Medical Informatics, Hôpital Européen Georges Pompidou, AP-HP, 20 Rue Leblanc, 75015 Paris, France; 2 INSERM, Centre de Recherche des Cordeliers, UMRS 1138, Université de Paris, Université Sorbonne Paris Cité, Paris, France

## Correction to: *Orphanet Journal of Rare Diseases* (2021) 16:139 10.1186/s13023-020-01639-7

Following the publication of the original article [1] the authors brought to our attention that their first and last names had unfortunately been interchanged.

The correct authors’ names are shown in the author list of this Correction and have already been updated in the original article.
